# OrdPrune-KD: An Ordinal-Consistency-Based Model Compression Framework for Diabetic Retinopathy Grading

**DOI:** 10.3390/s26123636

**Published:** 2026-06-07

**Authors:** Yuzhe Yan, Siqi Liang, Yifan Xia

**Affiliations:** School of Airspace Science and Engineering, Shandong University, Weihai 264209, China; yanyuzhe@mail.sdu.edu.cn (Y.Y.); liangsq@mail.sdu.edu.cn (S.L.)

**Keywords:** diabetic retinopathy grading, model compression, knowledge distillation, structured pruning, Earth Mover’s Distance (EMD), ordinal learning, deep learning, medical image analysis, lightweight neural networks, clinical decision support

## Abstract

This study proposes OrdPrune-KD, an ordinal-consistency-driven model compression framework that integrates grade-aware structured pruning with Earth Mover’s Distance (EMD)-based knowledge distillation for diabetic retinopathy (DR) grading. Unlike conventional approaches that only consider ordinal relationships at the loss level, the proposed method incorporates ordinal priors into both model compression and knowledge transfer stages. Extensive experiments on APTOS 2019, Messidor-2, and IDRiD demonstrate that the proposed framework achieves a favorable balance between model compactness and predictive performance. In particular, under a 77% parameter reduction, the student model achieves competitive performance relative to the teacher model in terms of QWK while maintaining strong high-risk sensitivity. Additional ablation studies and fairness-controlled comparisons confirm that the performance gains are primarily attributed to the proposed ordinal-aware design rather than output formulation differences. These results indicate that OrdPrune-KD provides an effective and deployable solution for lightweight DR grading systems.

## 1. Introduction

Diabetic retinopathy (DR) is a major microvascular problem caused by diabetes, and it affects many people around the world; it is also one of the main reasons why working-age adults lose their vision. Doctors usually look at color fundus photos to decide how serious the DR is, and they often divide it into five levels: Grade 0 means no DR and Grade 1 is mild, Grade 2 is moderate, Grade 3 is severe, and Grade 4 is proliferative DR. This way of grading follows a natural order, as described in references [1,2].

In the last few years, deep convolutional neural networks (or CNNs for short) have pushed forward the automatic grading of DR quite a lot; some large pre-trained models like EfficientNet [3] and ResNet [4] have shown diagnostic performance that is as good as, or even better than, human experts in several public competitions and benchmark datasets [5]; however, these high-performing models often bring a large number of parameters and high computational costs with them. For example, EfficientNet-B4 has about 17.55 million parameters and its inference latency is close to 9 ms, which means it depends heavily on GPU resources, and this dependency creates some practical problems in the following deployment scenarios:

Primary Care and Edge Deployment: In rural clinics with few resources, community health centers, or screening programs in developing countries, high-performance GPU servers are usually not available; large models also cannot run in real time on mobile devices or on embedded hardware, as noted in reference [6].

Mobile and Mini-Program Integration: Because internet-based healthcare is growing fast, the need for intelligent tools that help with diagnosis on lightweight platforms (like WeChat mini-programs and mobile apps) is becoming more and more urgent; if someone tries to put a large DR grading model directly onto these platforms, they will face long computation times and limits on memory.

Clinical Real-Time Feedback Requirements: In outpatient settings or remote consultation scenarios, eye doctors need the assistance system to finish looking at an image within a few seconds; lightweight models are necessary if we want to shorten the waiting time and make the doctor’s work go more smoothly [7,8].

Even though there has been some progress in making models smaller, there are still several key research gaps for DR grading. First, the common structured pruning methods, like magnitude-based uniform pruning and L1-norm pruning, treat all filters the same way; they do not take into account the ordinal relationships between the different severity levels, and this often leads to the unintended removal of those filters that are important for telling apart adjacent grades, for example Grade 2 vs. Grade 3 [9,10]. Second, the mainstream knowledge distillation methods, such as KL divergence, treat the five DR grades just like ordinary categories that have no order; they fail to make use of the distance information between grades, and they also have a hard time transferring the soft probability knowledge near the boundaries between grades [4,11]. Third, most of the existing lightweight models do not have a special focus on improving recall for the high-risk grades, which are Grades 3 and 4; this recall is arguably the most important safety measure in clinical screening because missing even one case of proliferative DR is much worse than getting a false positive. Fourth, there is not enough cross-dataset validation, especially on datasets with only a small number of samples, and this lack limits how useful these methods can be in practice.

The core issue is this: how can we fully leverage the ordinal nature of DR grading while substantially compressing model size and maintaining or even improving diagnostic sensitivity for high-risk patients? This question holds both theoretical value and urgent practical significance in medical image lightweighting.

For this reason, this study puts forward a framework called OrdPrune-KD; this framework takes the ordinal priors of DR grading and puts them into the whole-model compression pipeline in a more systematic way, and the main things we did in this paper are listed below:

1. Grade-Aware Structured Pruning: A joint scoring mechanism based on Taylor importance and ordinal discriminability is designed to protect critical filters that distinguish adjacent DR grades, avoiding the destruction of discriminative features by traditional pruning methods.
2. EMD Ordinal Knowledge Distillation: Earth Mover’s Distance (Wasserstein-1 distance) replaces standard KL divergence as the distillation loss, naturally respecting the ordinal structure of output distributions and more effectively transferring the teacher model’s soft label knowledge at grade boundaries.
3. Multi-scenario Experimental Validation: Systematic evaluation on three public DR benchmark datasets (APTOS 2019, Messidor-2, IDRiD), covering different data scales from large-scale (3662 images) to small-sample (533 images), validating the robustness and generalization capability of the framework.

Compared with existing work, the main innovation of this paper is not a single module improvement but an ordinal-consistent compression paradigm. This paradigm introduces ordinal information at both the model structure optimization stage (pruning) and the knowledge transfer stage (distillation). As a result, the model retains discriminability at grade boundaries during compression. This “full-pipeline consistency modeling” distinguishes our method from existing approaches that only add ordinal constraints at the loss function level.

## 2. Related Work

### 2.1. Deep Learning for Diabetic Retinopathy Grading

Since the landmark work by Gulshan et al. [5], deep learning-based automated grading of diabetic retinopathy (DR) has advanced rapidly. That study demonstrated ophthalmologist-level performance in DR detection using large-scale retinal image datasets. Subsequent efforts have continued to improve feature representation and grading robustness through developments in convolutional neural networks (CNNs), ordinal learning, and transformer-based architectures.

Early DR grading systems were largely built on conventional CNN backbones such as AlexNet, VGG, and ResNet [4]. Later, EfficientNet [3] improved parameter efficiency via a compound scaling strategy and has since become one of the most commonly used backbones for retinal image analysis. Several winning solutions on the APTOS 2019 benchmark, for instance, adopted EfficientNet-B4 or B5 combined with extensive data augmentation, model ensembling, and test-time augmentation to achieve state-of-the-art Quadratic Weighted Kappa (QWK) scores.

Lately, people have also tried using transformers for DR grading, and the results look quite good. For example, models based on Swin Transformer can capture long-range dependencies between retinal lesions quite well [12]. One study built on this idea, called Dual-SwinOrd [13], and combines features from Swin Transformer and PubMedCLIP and performs well on the APTOS 2019 benchmark. Some other researchers have brought in foundation-model-assisted frameworks or ordinal-aware transformer designs to make the grading more reliable and robust [14]. Vision Transformer (ViT) can also give strong feature representations for fine-grained retinal disease assessment [15]. However, these models do have a downside: they are heavy on both parameters and computation, which makes them less useful in situations where resources are tight, such as on mobile devices or edge platforms.

Several lightweight models for DR grading have been developed to help with deployment in situations where resources are limited; networks based on MobileNetV3 [16] and some lightweight attention designs give a good trade-off between efficiency and accuracy when it comes to analyzing retinal images. At the same time, some studies have looked into retinal screening systems that can be used with smartphone-assisted diagnosis and portable fundus cameras [8]. All these efforts have cut down on the computational cost and the model size, and this has made automated screening more practical in real-world settings. However, most of these lightweight methods focus a lot on efficiency, which means reducing the number of parameters or the computational load, but they do not pay much attention to the ordinal relationships that exist between the different DR severity levels.

Ordinal learning has become an important direction for DR grading, because unlike standard multi-class classification, the severity of DR has a natural order that goes from Grade 0 up to Grade 4. To make use of this order, some ordinal regression methods like CORAL and CORN break the grading task into a series of binary decisions, and these decisions respect the ranking, which helps to better capture how the disease progresses [2,17]. Other approaches have also been tried; these include label distribution learning [18], cumulative probability modeling, and uncertainty-aware ordinal frameworks, and all of these can help to handle the ambiguous boundaries that exist between grades and also improve how reliable the predictions are.

Recent work shows that using ordinal-aware learning strategies can help to improve both the grading performance and how well the model is calibrated; at the same time, careful evaluation also matters quite a lot. If data leakage happens because of improper threshold tuning or too much optimization on the validation set, this can give results that look too good and then do not hold up when used in actual clinical practice [19]. Knowledge distillation has also become a common way to compress large teacher networks into smaller student models for medical image analysis, but most existing distillation methods treat each disease category as if it were independent and do not preserve the ordinal relationships that exist between the adjacent severity levels [20]. Moreover, ordinal information is usually only added at the prediction stage or the loss stage, while keeping that ordinal information during model compression, and lightweight deployment has not received nearly as much attention.

Overall, despite substantial advances in DR grading accuracy, existing high-performance methods still suffer from large model size and high computational complexity. Furthermore, current lightweight DR systems rarely integrate ordinal-aware optimization into the full compression pipeline.

### 2.2. Model Compression and Structured Pruning

Model compression is key to running deep neural networks on medical devices with limited resources. Current compression methods include pruning, quantization, low-rank decomposition, neural architecture search (NAS), and knowledge distillation.

Pruning is a popular way to cut down computational cost and memory use. Early work often judged a parameter’s importance by its weight magnitude. Han et al. [21], for instance, showed that sparse pruning plus weight sharing could greatly reduce model redundancy. But the irregular sparsity pattern from these methods makes them hard to speed up on general-purpose hardware.

To get around this, structured pruning removes whole filters, channels, or network blocks while keeping the architecture hardware-friendly. Well-known examples include Taylor expansion-based filter importance estimation from Molchanov et al. [9], channel selection using BN scaling factors in Network Slimming [10], and filter-level pruning strategies from Li et al. [22]. These methods run inference more efficiently and work better in real-world deployment.

As practical deployment becomes more important, researchers have started looking into pruning strategies that are aware of hardware and aimed at real-world use [23,24]. Unlike older methods that mainly focus on cutting down FLOPs, these approaches take into account the hardware’s characteristics and the inference latency during compression. Later work looked at latency-aware structured pruning and optimization for edge devices, and it reported good trade-offs between accuracy and efficiency under real-world deployment conditions [25,26].

Structured pruning has also worked well in several medical imaging applications, including retinal disease screening [6], chest X-ray analysis [27], and skin lesion classification [28]. In these cases, the compressed models cut down on the computational needs while still keeping the diagnostic performance competitive. However, the existing pruning frameworks are mostly designed for standard classification tasks, and they rarely take into account the ordinal relationships that exist in disease severity grading.

Despite these advances, the existing pruning methods are generally task-agnostic, and they treat all classes the same way during the importance estimation step. But in ordinal medical classification tasks like DR grading, different filters do not all contribute equally to telling apart the adjacent severity levels. Those filters that are sensitive to clinically important grade transitions, for example Grade 2 vs. Grade 3, should receive stronger protection during the compression process; however, the existing pruning methods rarely consider this kind of ordinal discriminability when they do structured model optimization.

### 2.3. Knowledge Distillation and Ordinal Distillation

Knowledge distillation (KD) was first introduced by Hinton et al. [29], referring to a process where a small student model learns from the soft probability outputs of a larger teacher model. FitNets [30] later added intermediate feature alignment to improve how information is passed from teacher to student. Other KD methods have since explored attention transfer [31], relational distillation [32], contrastive distillation [33], and more recently, distillation frameworks built specifically for medical imaging under limited resources [25,34].

Knowledge distillation has become a common way to compress models in medical image analysis; it has been used in lightweight retinal diagnosis systems [34,35], in retinal disease screening frameworks [36], and in other healthcare settings where computing resources are limited [20,37]. By transferring knowledge from a large teacher network over to a small student model, distillation can often boost the student’s predictive performance and generalization, especially when the amount of annotated medical data is small [38].

Most of the current distillation methods use Kullback–Leibler (KL) divergence to align the output distributions of the teacher and student networks; this works fine for standard classification tasks, but KL divergence does not take into account the ordered relationships that exist between the classes, and that matters for DR grading because the categories follow a clear progression in terms of severity. Confusing Grade 2 with Grade 3, for example, is a much smaller clinical mistake than confusing Grade 2 with Grade 0; however, KL-based optimization does not directly capture this ordinal distance, and this may hurt how well the knowledge gets transferred for ordinal prediction tasks.

A few recent studies have looked into using Wasserstein-distance-based learning and label distribution matching to get around this issue [37,39,40]. Earth Mover’s Distance (EMD) naturally captures the cost of moving probability mass between classes, and it has worked well for age estimation [41], for medical risk prediction, and for ordinal label distribution learning [42]. Wasserstein-distance-guided ordinal representation learning [43] and ordinal-aware Wasserstein methods [44] have also shown better robustness when the boundaries between classes are not very clear. More recently, some researchers combined optimal transport theory with ordinal representation learning, and they found that this helps to preserve the ordering information between classes during the knowledge transfer process.

Nevertheless, existing ordinal distillation studies mainly focus on output-level supervision and do not jointly integrate ordinal constraints into both model compression and knowledge transfer. Furthermore, the interaction between ordinal-aware pruning and ordinal-aware distillation remains largely unexplored in medical imaging tasks.

## 3. Methods

**Figure 1:**
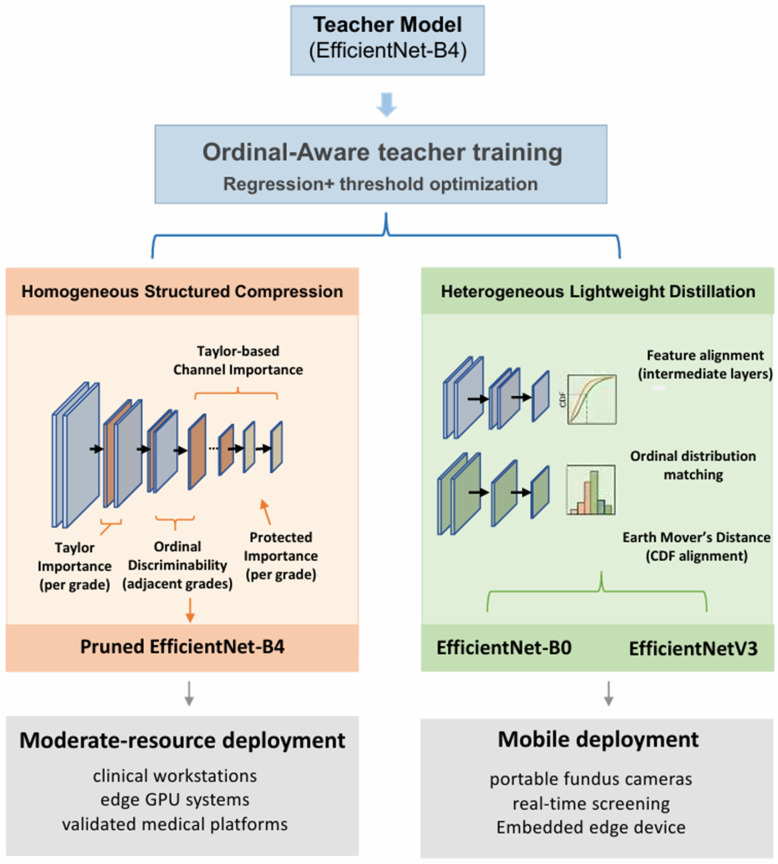
OrdPrune-KD framework diagram. The framework leverages ordinal information from DR severity grades (0–4) to guide the structured pruning and EMD-based knowledge distillation process, thereby obtaining a student model with both compact structure and preserved ordinal prediction capability.

The proposed OrdPrune-KD framework aims to achieve lightweight and ordinal-consistent diabetic retinopathy grading through a three-phase compression pipeline, as illustrated in Figure 1.

In Phase 1, an EfficientNet-B4 teacher model gets trained using ordinal regression optimization, and this is done to build a reference network that has high capacity and is aware of ordinal information. Then, based on this trained teacher, Phase 2 carries out grade-aware structured pruning to get homogeneous compressed models while still keeping the ordinal discriminative features that matter clinically; this stage is mainly aimed at deployment situations where architectural consistency and compatibility with existing EfficientNet-based systems are needed.

To make deployment more flexible and to improve computational efficiency even further, Phase 3 brings in EMD-based ordinal knowledge distillation for both homogeneous and heterogeneous student models. Unlike what happens in Phase 2, the heterogeneous lightweight students, like EfficientNet-B0 and MobileNetV3, get distilled directly from the full teacher model without needing any intermediate pruning step. So, homogeneous pruning and heterogeneous lightweight distillation should be seen as compression strategies that go together and are meant for different deployment constraints, rather than as steps that have to follow one another in a strict order.

Compared with the more conventional ways of compressing models, which mostly focus on reducing the number of parameters, the framework we are proposing here puts ordinal information into both the pruning stage and the distillation stage in a clear way, and this helps the compressed student models to better preserve the grade relationships that are important from a clinical point of view.

### 3.1. Phase 1: Teacher Model Training

EfficientNet-B4 [3] was adopted as the teacher backbone due to its favorable balance between representational capacity and computational efficiency in medical image classification tasks. The teacher model was trained using a regression-based ordinal prediction framework, where a single continuous output was optimized to reflect the severity progression of diabetic retinopathy grades.

To improve ordinal consistency, the training objective combines SmoothL1 regression loss with ordinal rank regularization. In addition, stochastic weight averaging (SWA), Mixup/CutMix augmentation, and class-rebalanced sampling were employed to improve generalization performance and alleviate class imbalance.

#### 3.1.1. Loss Function Design

The training loss adopts a combined form of SmoothL1 regression loss and ordinal rank regularization:

(1) $$L_{teacher}=L_{SmoothL1}\left(\hat{y},\;y\right)+\lambda \cdot L_{OrdRank}\left(\hat{y},\;y\right).$$

where $\hat{y}$ represents the continuous output predicted by the model, *y* represents the true DR grade, and λ represents the regularization weight coefficient. The ordinal rank regularization penalizes prediction pairs that violate the monotonic relationship between grades, encouraging model outputs to remain consistent with the five-level ordinal structure of DR, thereby encoding inter-class ordering information in the continuous output space [2].

#### 3.1.2. Training Strategy

The learning rate schedule uses cosine annealing with a 3-epoch linear warmup. Stochastic weight averaging (SWA) [45] is applied in the last 20% of training epochs, improving generalization performance by averaging multiple weight checkpoints along the training trajectory, enabling the model to converge to a wider loss basin.

For data augmentation, a mixed strategy of Mixup [46] and CutMix [47] is introduced: Mixup performs convex combination interpolation on training samples and their labels, while CutMix replaces a rectangular region of one image with the corresponding region of another image, simultaneously mixing the labels of both. These label-softening strategies effectively improve the model’s robustness to ambiguous samples near DR grade boundaries. To address the severe class imbalance in the APTOS 2019 dataset (Grade 0 at 49.3%, Grade 3 at only 7.3%), an inverse-frequency-based weighted random sampling strategy is adopted to ensure balanced presentation of each grade’s samples in training batches.

Beyond augmentation and sampling techniques, the clinical translation of automated DR grading systems has been substantially advanced by large-scale validation and regulatory approval efforts. Abràmoff et al. [48] conducted a pivotal trial of an autonomous AI-based DR diagnostic system across 10 primary care sites, achieving 87.2% sensitivity and 90.7% specificity for detecting more-than-mild DR, which led to the first FDA authorization of a fully autonomous AI diagnostic system in medicine. Similarly, Li et al. [49] developed and validated a deep learning algorithm for vision-threatening referable DR detection using 106,244 retinal images, demonstrating robust performance (AUC 0.989 internally and 0.955 in external multi-ethnic validation) and highlighting the potential of automated systems to improve DR screening accessibility in community settings. These clinical validation studies underscore the practical importance of developing accurate, trustworthy, and deployable DR grading systems—aligning with the overarching goal of the proposed OrdPrune-KD compression framework.

### 3.2. Phase 2: Homogeneous Structured Compression Route

Standard structured pruning methods perform undifferentiated importance ranking of all filters using magnitude or global Taylor expansion scores [9], ignoring the differentiated dependence of different grades on different filters in DR grading tasks. The grade-aware pruning mechanism proposed in this paper explicitly protects the filters most critical for ordinal grade discrimination during compression through a three-step joint scoring process.

#### 3.2.1. Step 1: Taylor Importance Matrix

For the *c*-th filter in the *l*-th layer of the network, Taylor importance scores are computed separately for each DR severity grade k∈{0, 1, 2, 3, 4}:

(2) $$I_{k,c}^{\left(l\right)}={∥\frac{\partial L_{k}}{\partial W_{c}^{\left(l\right)}}⊙W_{c}^{\left(l\right)}∥}_{\mathrm{mean}\;\mathrm{over}\;(C_{in},H,W)}.$$

where $L_{k}$ is the grade *k* proxy loss defined with Sigmoid activation, computed by backpropagating gradients corresponding to each grade over training batches. Unlike the original global Taylor importance method, this paper independently computes importance scores for each grade, constructing an importance matrix of shape $K\times C^{\left(l\right)}$, capturing the specific contribution of each filter to each DR grade, providing a basis for subsequent ordinal discriminability evaluation.

#### 3.2.2. Step 2: Ordinal Discriminability Score

Based on the importance matrix obtained in Step 1, the discriminative ability of each filter for adjacent grades is computed:

(3) $$D_{c}^{\left(l\right)}=\sum_{k=0}^{K-2}\left|I_{k+1,c}^{\left(l\right)}-I_{k,c}^{\left(l\right)}\right|.$$

where $D_{c}^{\left(l\right)}$ represents the ordinal discriminability score of the *c*-th filter and *K* represents the number of grades. This score quantifies the magnitude of difference in importance between adjacent DR grades for the filter: if a filter has significantly different activation responses between adjacent grade pairs (such as Grade 2 and Grade 3), its *D* value is larger, indicating that the filter has a strong specialized ability in distinguishing ordinal grades and should be prioritized for retention during pruning. Compared to methods relying solely on global importance, the ordinal discriminability score can identify those filters that are sensitive to grade boundaries but not particularly prominent in global importance, thereby better maintaining the model’s ability to distinguish high-risk grades after compression.

#### 3.2.3. Step 3: Protection Importance Score

Combining the base importance with the discriminability protection factor yields the final pruning score:

(4) $${\mathrm{FinalImp}}_{c}^{\left(l\right)}=I_{c}^{\left(l\right)}\cdot \left(1+\gamma \cdot {\hat{D}}_{c}^{\left(l\right)}\right),\;\gamma =2.0.$$

where ${\hat{D}}_{c}^{\left(l\right)}$ is the min-max normalized discriminability score, $I_{c}^{\left(l\right)}=\frac{1}{K}\sum_{k}I_{k,c}^{\left(l\right)}$ is the mean importance across grades, and γ=2.0 is the discriminability protection coefficient. The multiplicative structure ensures that filters with high ordinal discriminability receive proportional amplification in the final score, prioritizing the retention of feature extractors most discriminative at grade boundaries while ensuring the overall compression rate.

#### 3.2.4. Iterative Pruning Strategy

The pruning process is performed iteratively in 5 steps, with fine-tuning epochs (learning rate 1$\times \;10^{-4}$) inserted between each step to progressively recover performance loss caused by pruning in the current step. This progressive strategy has been proven superior to one-time large-scale pruning, enabling the network to re-adapt to new parameter distributions as capacity is gradually reduced.

In this study, the terms “Pruned 30%”, “Pruned 50%”, and “Pruned 70%” refer to the target channel pruning ratios applied during structured pruning. Due to architectural dependencies and layer-wise parameter redistribution after pruning, the actual parameter reduction ratios relative to the original teacher model may differ from the target pruning ratios. Therefore, the “Compress (%)” values reported in the experimental tables denote the actual parameter reduction relative to the original EfficientNet-B4 teacher model.

### 3.3. Phase 3: Heterogeneous Lightweight Deployment Route

The student network obtained by grade-aware pruning then undergoes knowledge distillation training. Unlike the teacher model’s regression output, the distilled student model uses a 5-dimensional Softmax classification output, completing final grade prediction through expected value calculation combined with validation set threshold optimization.

#### 3.3.1. EMD Distillation Loss

Standard knowledge distillation measures the difference between teacher and student output distributions using KL divergence, but KL divergence treats each class as unordered independent probability masses, unable to perceive the ordinal distance relationships between DR grades. This paper replaces KL divergence with Earth Mover’s Distance (EMD), i.e., Wasserstein-1 distance [40], as the distillation loss:

(5) $$L_{\mathrm{EMD}}\left(p,q\right)=\sum_{k=1}^{K}\left|{\mathrm{CDF}}_{k}\left(p\right)-{\mathrm{CDF}}_{k}\left(q\right)\right|.$$

where ${\mathrm{CDF}}_{k}\left(p\right)=\sum_{j=0}^{k-1}p_{j}$ is the cumulative distribution function, and *p* and *q* are the output probability distributions of teacher and student, respectively (Softmax after temperature scaling). EMD naturally considers the “transportation cost” between classes by computing the area difference between two distributions in the cumulative distribution function space: placing prediction probability incorrectly from Grade 2 to adjacent Grade 3 incurs a much smaller EMD penalty than placing it at distant Grade 0, reflecting the inherent distance structure of ordinal classification. In contrast, KL divergence applies nearly identical penalties to both deviations, and is unable to distinguish the clinical severity of grade deviations.

#### 3.3.2. Complete Distillation Loss Function

The total distillation loss is a weighted combination of three components:

(6) $$L_{\mathrm{total}}=T^{2}\cdot L_{\mathrm{EMD}}\left(\sigma \left(\frac{z_{S}}{T}\right),\sigma \left(\frac{z_{T}}{T}\right)\right)+\alpha \cdot L_{\mathrm{OrdCE}}\left(z_{S},y\right)+\beta \cdot L_{\mathrm{feat}}.$$

The meanings of each term are as follows: The first term $T^{2}\cdot L_{\mathrm{EMD}}$ is the ordinal soft-label distillation loss, where T=4.0 is the temperature coefficient, and the $T^{2}$ factor compensates for the gradient scale changes caused by temperature scaling; $z_{S}$ and $z_{T}$ are the predicted logits of the student and teacher, respectively. The second term $L_{\mathrm{OrdCE}}$ is the ordinal cross-entropy loss, maintaining the student model’s basic classification capability under hard-label supervision. The third term $L_{\mathrm{feat}}$ is the intermediate layer feature alignment loss, applying MSE constraints on L2-normalized features from 3 intermediate layers through 1 × 1 convolutional adapters, guiding the student to reproduce the teacher’s intermediate representation structure. The weight coefficients are set as α=0.5, β=0.3, and the coefficient corresponding to $L_{\mathrm{feat}}$ is 0.2, summing to 1.0.

#### 3.3.3. Heterogeneous Distillation

In addition to using the same-specification pruned network as the student (homogeneous distillation), this framework also supports transferring teacher knowledge to independently designed efficient compact architectures (heterogeneous distillation), specifically EfficientNet-B0 and MobileNetV3-Large [16]. In heterogeneous distillation, the feature alignment loss bridges the gap in intermediate feature dimensions between teacher and student networks through learnable 1 × 1 convolutional adapter layers, enabling target architectures of different capacity specifications to benefit from the teacher’s ordinal knowledge.

Unlike homogeneous pruning-based compression, heterogeneous distillation does not require preserving the original EfficientNet-B4 architecture. Instead, it enables direct transfer of ordinal knowledge to lightweight backbones optimized for deployment efficiency. Therefore, heterogeneous distillation is particularly suitable for resource-constrained edge devices and real-time screening systems, where low latency and reduced memory footprint are more critical than architectural consistency.

### 3.4. Inference Phase: Threshold Optimization and Leak-Free Evaluation

The distilled student model outputs a 5-dimensional Softmax probability distribution, obtaining a continuous ordinal prediction value through expected value calculation:

(7) $${\hat{y}}_{\exp}=\sum_{k=0}^{K-1}k\cdot p_{k}.$$

where $p_{k}$ represents the *k*-th class probability of the model’s prediction, and ${\hat{y}}_{\exp}$ represents the continuous expected prediction value. Subsequently, the optimal grading threshold vector $\theta^{*}\in R^{K-1}$ is searched on the validation set through the Nelder–Mead simplex algorithm [50], with the objective of maximizing the validation set QWK:

(8) $$\theta^{*}=\arg\min_{\theta }\left[-QWK\left(y_{\mathrm{val}},\mathrm{digitize}\left(\hat{y_{\mathrm{val}}^{\exp}},\theta \right)\right)\right].$$

The final prediction is ${\hat{y}}_{\mathrm{test}}=\mathrm{digitize}\left({\hat{y}}_{\exp}^{\mathrm{test}},\theta^{*}\right)$, where the optimized threshold $\theta^{*}$ is directly applied to the test set without access to test set labels throughout. This leak-free evaluation protocol prevents threshold overfitting to the test set distribution, ensuring all reported metrics reflect true generalization performance [19]. The teacher model’s regression inference also uses validation set-calibrated thresholds, maintaining consistent evaluation standards with the student model.

## 4. Experiments

### 4.1. Experimental Setup

#### 4.1.1. Datasets

This study is evaluated on three public diabetic retinopathy benchmark datasets, covering different data scale scenarios from large-scale to small-sample, to fully validate the robustness and generalization capability of the framework.The detailed statistics and train/validation/test splits of these three benchmark datasets are summarized in Table 1.

All three datasets contain color fundus photographs annotated with five-level DR severity: Grade 0 (no DR), Grade 1 (mild), Grade 2 (moderate), Grade 3 (severe), and Grade 4 (proliferative DR). APTOS 2019 has a significant class imbalance problem, with Grade 0 samples accounting for 49.3% and Grade 3 only 7.3%, which is the main motivation for adopting weighted sampling strategies and high-risk sensitivity metrics in this study. IDRiD contains only 533 images, making it the most challenging low-data evaluation scenario among the three.

#### 4.1.2. Preprocessing and Data Augmentation

All images uniformly adopt the Ben Graham preprocessing pipeline [51]: circular cropping first, then resizing to 512 × 512 pixels, and finally contrast normalization through addWeighted with Gaussian blur (θ=10). The training phase uses the Albumentations library [52] to implement various augmentation strategies, including random 90° rotation, horizontal/vertical flipping, affine transformations (translation amplitude 10%, scale ratio 0.85–1.15, rotation angle ±45°), Gaussian/median blur, color jitter, and CoarseDropout. Additionally, Mixup and CutMix mixed augmentation are applied. In terms of input resolution, APTOS 2019 uses 380 × 380 pixels, while Messidor-2 and IDRiD use 224 × 224 pixels. For class imbalance, an inverse-frequency-based weighted random sampling strategy is adopted.

All experiments were implemented using PyTorch 2.8.0 and Python 3.12 under the CUDA 12.8 environment. Training and evaluation were performed on a workstation equipped with a single NVIDIA RTX 4090 GPU with 24 GB memory. Unless otherwise specified, the batch size was set to 32 for all experiments.

#### 4.1.3. Evaluation Metrics

This study adopts the following four types of metrics for the comprehensive evaluation of model performance:

1. Quadratic Weighted Kappa (QWK). The standard primary evaluation metric for DR grading competitions:
  (9) $$\omega_{i,j}=\frac{{\left(i-j\right)}^{2}}{{\left(N-1\right)}^{2}},$$
  (10) $$\kappa =1-\frac{\sum_{i=1}^{N}\sum_{j=1}^{N}w_{i,j}O_{i,j}}{\sum_{i=1}^{N}\sum_{j=1}^{N}w_{i,j}E_{i,j}}.$$
  where $\omega_{i,j}$ represents the weight between grades *i* and *j*, $O_{i,j}$ represents observed frequencies, $E_{i,j}$ represents expected frequencies, and *N* is the number of grades.
2. Per-class AUC (mAUC). ROC-AUC for each grade (G0–G4) is computed using the One-vs.-Rest strategy and averaged.
3. High-Risk Sensitivity (HR Sens.). Combined recall rate for Grade 3 and Grade 4 (severe and proliferative DR), directly reflecting clinical screening safety.
4. Efficiency Metrics: Including number of trainable parameters (M), floating-point operations for forward propagation (FLOPs, G), and GPU inference latency (ms, mean of 50 runs with 10 warm-up runs).

Threshold Optimization Note: All QWK-related threshold optimization (expected value calculation combined with Nelder–Mead optimization) is strictly performed on the validation set, and the optimized thresholds are then applied to the test set to prevent data leakage.

For all reported results, model training and evaluation were repeated five times using different random seeds. Performance values are reported as mean ± standard deviation.

### 4.2. Single-Dataset Experimental Results

#### 4.2.1. APTOS 2019 Results

The core findings of the APTOS experiments are as follows: First, the distilled student model achieves comparable or slightly improved QWK scores under the current evaluation protocol. Specifically, the EfficientNet-B0 student model achieves a QWK of 0.9194, demonstrating competitive performance relative to the teacher model (QWK = 0.9034) while reducing the parameter count by approximately 77%. Second, distillation consistently improves pruned model performance: distillation + Pruned 30% improves from 0.8996 to 0.9154 (+1.6%); distillation + Pruned 50% improves from 0.8972 to 0.9158 (+1.9%). Third, pruning alone has a limited impact on QWK: at a 50% compression rate, QWK drops only 0.7% (0.9034→0.8972). Fourth, real-time inference is achieved: MobileNetV3 reaches 3.6 ms inference latency (2.5× speedup) with 85.9% FLOP reduction.The comprehensive experimental results, including performance metrics, compression rates, and inference latencies, are summarized in Table 2.

#### 4.2.2. Messidor-2 Results

The Messidor-2 experiments reveal several important patterns. Heterogeneous distillation significantly outperforms homogeneous distillation: Distill→B0 (QWK = 0.7778) and MNV3 (0.7730) substantially surpass homogeneous distillation + Pruned 30% (0.7139), approaching teacher-level performance (0.8017). In terms of high-risk sensitivity, Distill→B0 maintains an extremely high level of 0.939, with a smaller gap from the teacher (0.9697). Additionally, Messidor-2 is more sensitive to aggressive pruning—QWK plummets to 0.1216 at 70% pruning, indicating that this dataset has higher requirements for model capacity.The comprehensive experimental results, including performance metrics, compression rates, and inference latencies, are summarized in Table 3.

#### 4.2.3. IDRiD Results

IDRiD experiments demonstrate that OrdPrune-KD remains highly effective even with only 319 training images. Distillation + Pruned 30% achieves a QWK of 0.7585, which is marginally higher than the teacher model (QWK = 0.7364) under the current evaluation protocol, while mAUC reaches the highest value of 0.8118, and high-risk sensitivity is maintained at 0.938, meaning only about 6.2% of severe cases would be missed. Notably, on IDRiD, homogeneous distillation (Pruned 30%) outperforms heterogeneous distillation (B0), contrary to the conclusion on APTOS, suggesting that when the data volume is small, a smaller model capacity better matches the limited data.The comprehensive experimental results, including performance metrics, compression rates, and inference latencies, are summarized in Table 4.

### 4.3. Cross-Dataset Comparative Analysis

**Figure 2:**
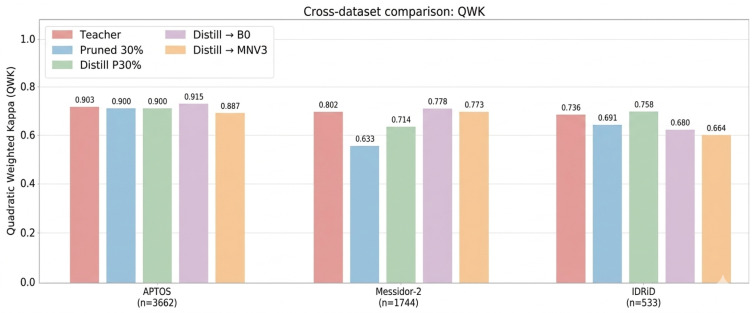
QWK comparison across three datasets for key model configurations.

**Figure 3:**
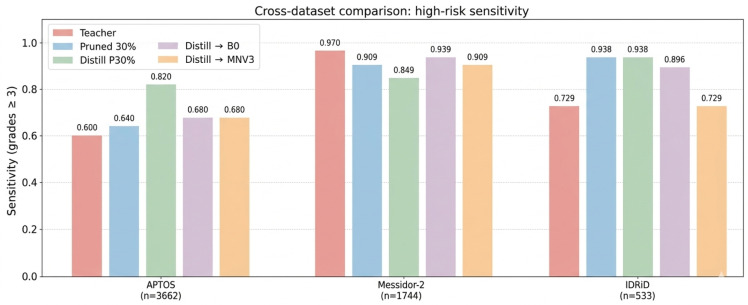
High-risk sensitivity (Grade 3 + 4 recall) across datasets. Distilled models consistently maintain strong clinical safety.

Cross-dataset analysis reveals several patterns of great significance. First, consistency of distillation benefits: on two of the three datasets (APTOS and IDRiD), the optimal distilled student achieves QWK scores that are comparable to, or marginally higher than, those of the teacher model under the current evaluation protocol; on Messidor-2, the B0 student achieves a QWK only 2.4% lower than the teacher while reducing parameters by 77%. Second, absolute performance positively correlates with data scale: QWK increases with training set size (APTOS: 0.919 > Messidor-2: 0.778 > IDRiD: 0.759), consistent with the general pattern of deep learning. Third, optimal architecture selection is dataset-dependent: on larger datasets (APTOS), heterogeneous B0 distillation performs best; on smaller datasets (IDRiD), homogeneous Pruned 30% distillation performs better, possibly due to better matching between smaller model capacity and limited data volume. Fourth, high-risk sensitivity is robust: distilled models maintain strong high-risk sensitivity (≥0.68) on all datasets, with Distill + Pruned 30% reaching 0.938 on IDRiD. These observations further suggest that homogeneous compression and heterogeneous distillation should not be viewed as competing stages, but rather as complementary deployment strategies under different hardware and system constraints. The detailed cross-dataset evaluation results and performance comparisons are summarized in Table 5, while the comprehensive visual comparisons for QWK scores and high-risk sensitivity across different model configurations are illustrated in Figure 2 and Figure 3, respectively.

#### 4.3.1. Distillation Recovery Effect and Efficiency Analysis

**Figure 4:**
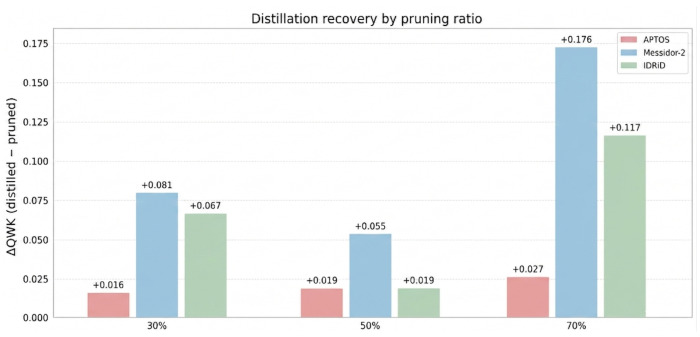
Distillation recovery: QWK improvement from pruned to distilled models. Positive values indicate successful knowledge recovery through EMD distillation.

Figure 4 shows QWK gains from pure pruning to post-distillation, quantifying the knowledge recovery effect of EMD distillation. As shown, EMD ordinal distillation brings positive QWK gains (ΔQWK > 0) across all combinations of compression rates and datasets, validating the universal effectiveness of knowledge recovery. At the aggressive compression rate of 70%, the recovery effect of distillation is particularly significant, with ΔQWK as high as +0.176 on Messidor-2 and +0.117 on IDRiD, indicating that when pruning causes greater capability loss, distillation can play a stronger compensatory role.

#### 4.3.2. Parameter–Performance Trade-Off (Pareto Analysis)

**Figure 5:**
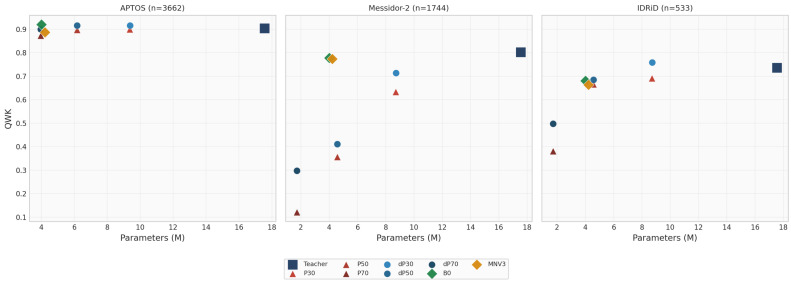
Parameters vs. QWK trade-off on each dataset. Diamond markers (heterogeneous distillation) achieve the best efficiency on larger datasets.

Figure 5 displays the Pareto frontier of each model on three datasets, with parameter count as the x-axis and QWK as the y-axis, intuitively showing the trade-off between compression and accuracy. Pareto analysis shows that heterogeneous distillation models (star markers) occupy Pareto-optimal positions on larger datasets (APTOS, Messidor-2), achieving the best parameter–performance trade-off at approximately 4 M parameters. Homogeneous distillation models (circle markers) consistently outperform pure pruning models (triangle markers) at the same parameter count, validating the universal effectiveness of knowledge distillation.

### 4.4. Fairness-Controlled Comparison

Even under identical five-class classification head, expectation decoding, and threshold calibration protocol, the proposed OrdPrune-KD framework consistently improves QWK by 5–6 percentage points over scratch-trained lightweight models. This confirms that the performance gains arise from the proposed ordinal-aware compression and distillation strategy rather than differences in output formulation.The corresponding experimental results and ablation comparisons are detailed in Table 6.

### 4.5. Ablation Study

To systematically evaluate the independent contribution of each component of OrdPrune-KD, this study conducts stepwise ablation experiments on the APTOS 2019 dataset with a fixed 30% target pruning ratio. Groups A–C use regression output with threshold optimization; Groups D–E use classification heads, performing inference through expected value calculation combined with Nelder–Mead validation set optimization.The quantitative ablation results for each model configuration are summarized in Table 7.

The detailed analysis of each ablation group’s results is as follows:

- B vs. A (Uniform Pruning vs. Teacher): Despite reducing parameters by approximately 50% (from 17.55 M to 8.71 M), the model’s QWK (0.903→0.907) remains basically stable, but both Acc (0.803→0.776) and High-Risk Sensitivity (HR Sens.) (0.600→0.580) decline. This indicates that traditional uniform pruning can effectively compress model size but results in some loss in critical category discriminability.
- C vs. B (Grade-Aware Pruning vs. Uniform Pruning): At the same parameter scale (8.71 M), Acc significantly improves from 0.776 to 0.801, and HR Sens. dramatically improves from 0.580 to 0.720. This indicates that this method can more specifically retain important features related to ordinal labels, with better adaptability for clinically critical grading tasks.
- D vs. C (KL Distillation vs. No Distillation): KL distillation improves mAUC from 0.900 to 0.920, indicating enhanced model performance in overall probability distribution fitting. However, both Acc (0.801→0.754) and QWK (0.908→0.888) decline, and HR Sens. shows no improvement (remains at 0.720). This indicates that distribution matching-based distillation focuses more on global information alignment while having limited improvement in ordinal relationship and critical category discriminability.
- E vs. D (EMD Distillation vs. KL Distillation): Replacing the distillation loss from KL divergence to EMD improves high-risk sensitivity from 0.720 to 0.760 (+5.6 percentage points), while mAUC somewhat declines (0.920→0.904). This result indicates that EMD’s ordinal-aware characteristic is more conducive to retaining discriminative information at grade boundaries, directly improving the recognition capability for the most clinically critical high-risk grades. The trade-off between the two distillation methods reflects the inherent tension between ordinal knowledge transfer and average class discrimination, with EMD distillation having greater advantages in clinically application-oriented metrics (high-risk sensitivity). Synthesizing the ablation study results, each component in the OrdPrune-KD framework significantly contributes to the final performance: grade-aware pruning provides a more discriminative initial network structure for the distillation phase, while EMD ordinal distillation effectively recovers compression losses and strengthens perception of high-risk grades, with both working synergistically to achieve dual improvements in performance and efficiency.

### 4.6. Comparison with Published Methods

In addition to conventional DR grading methods, Table 8 also includes recent lightweight and efficiency-oriented models to evaluate the proposed framework from both diagnostic performance and deployment perspectives.

From Table 8, it can be seen that the proposed OrdPrune-KD framework demonstrates competitive performance on the APTOS 2019 benchmark for five-class diabetic retinopathy (DR) grading tasks. Several key conclusions can be drawn.

First, in the single-model setting, the teacher model based on EfficientNet-B4 achieves a QWK score of 0.903, which is comparable to or slightly better than recent ordinal regression methods (QWK ≈ 0.899). This result confirms the effectiveness of EfficientNet-based architectures in DR grading tasks, even without relying on ensemble strategies or multimodal inputs.

Second, the distilled student model (→B0) achieves competitive performance, achieving a QWK value of 0.919 while maintaining a compact architecture (4.01 million parameters). Compared to representative single-model methods such as MSA-Net (QWK = 0.894) and standard ordinal regression (QWK = 0.899), the proposed method demonstrates clear advantages. Notably, its performance approaches more complex solutions such as ensemble-based methods (QWK = 0.936) and vision-language dual-head models (QWK = 0.937), while requiring significantly fewer computational resources.

Third, the core contribution of this work lies not in achieving the absolute highest QWK score, but in achieving an effective balance between accuracy and model efficiency. Achieving near state-of-the-art performance with a lightweight model highlights its practical value, especially for deployment in resource-constrained environments such as embedded medical devices and edge computing systems.

Although direct comparisons with recent pruning-based and knowledge-distillation-based DR compression frameworks are limited due to differences in evaluation protocols and public availability, the proposed OrdPrune-KD achieves a competitive balance between diagnostic performance and compression ratio.

### 4.7. Efficiency Analysis

Table 9 provides a detailed comparison of the computational efficiency of different model variants, highlighting the advantages of the proposed heterogeneous knowledge distillation and pruning strategies.

First, in terms of compression efficiency, distilled student models (e.g., →B0 and →MobileNetV3) achieve substantial reductions in both parameters and FLOPs. Specifically, the →B0 model reduces parameters and FLOPs by approximately 77.2% and 74.5%, respectively, while maintaining a QWK score above 0.887. The →MobileNetV3 variant further reduces FLOPs by as much as 85.9%, demonstrating the effectiveness of heterogeneous distillation in minimizing computational costs without significantly degrading performance.

Second, in terms of inference latency, lightweight student models demonstrate significant advantages. The →MobileNetV3 model has an inference latency of only 3.6 ms, achieving a 2.47× speedup compared to the teacher model. This efficiency level is particularly suitable for real-time DR screening scenarios, such as portable fundus cameras and edge AI diagnostic systems. From a deployment perspective, homogeneous pruning and heterogeneous lightweight distillation target different engineering requirements. Pruned EfficientNet-B4 models preserve architectural consistency and are easier to integrate into existing medical AI systems already optimized for the EfficientNet-B4 backbone. In contrast, heterogeneous student models prioritize inference efficiency and deployment flexibility for mobile and embedded platforms.

Third, from an engineering deployment perspective, the proposed method successfully balances model compactness, computational efficiency, and diagnostic performance. Importantly, it achieves rapid and resource-efficient inference while maintaining clinically relevant performance (e.g., high-risk sensitivity ≥ 0.68). These characteristics are highly consistent with the requirements of deployable, real-time, and versatile intelligent sensing systems in medical applications.

## 5. Discussion

### 5.1. Why Compressed Students Can Achieve Competitive Performance Relative to the Teacher

The observation that some compressed student models get results that are as good as, or even a little better than, the teacher model can be explained by a few things.

One possible reason is the way the predictions are set up. The teacher network uses an ordinal regression framework with threshold-based decoding, while the student models use a five-class probability output plus expected-value decoding and threshold optimization; so, some of the difference in performance may come from differences in the inference and decoding steps, not just from the model compression itself.

Another thing that plays a role is the regularization effect that comes with model compression. Compared to the larger EfficientNet-B4 teacher, the compressed student networks have a lower capacity for representation, and this can help reduce overfitting and make the model work better on smaller medical imaging datasets.

Also, the proposed EMD-based distillation framework gives smoother supervision information by using temperature-scaled soft targets. This kind of supervision may be especially helpful for retinal images that fall near the boundaries between adjacent DR grades, because hard-label optimization is often sensitive to uncertainty in the annotations and the confusion between grades.

It is also worth noting that the improvements seen in QWK are quite small. How big these improvements are may depend on the dataset’s characteristics, the output formats, and the evaluation settings; therefore, these results should be seen as evidence that ordinal-aware compressed models can keep competitive diagnostic performance under real-world deployment constraints, rather than as proof that compressed models always do better than larger teacher networks across different tasks and settings.

Furthermore, although a classification-based teacher baseline was not included in the current study, the fairness-controlled comparison against scratch-trained lightweight models under identical classification heads, expectation decoding, and threshold calibration protocols provides additional evidence that the observed performance gains are primarily attributable to the proposed ordinal-aware compression and distillation strategy rather than differences in output formulation alone.

### 5.2. Cross-Dataset Generalization

OrdPrune-KD’s consistent effectiveness across three datasets with vastly different scales (533 to 3662 images) confirms its robustness. Homogeneous pruning-based compression and heterogeneous distillation-based compression exhibit complementary advantages under different deployment constraints. Structured pruning is preferable when architectural consistency, system compatibility, and stable integration are required, whereas heterogeneous lightweight distillation is more suitable for highly resource-constrained edge deployment scenarios requiring lower latency and reduced memory usage.

### 5.3. Architectural Generalizability and Limitations

Even though OrdPrune-KD got stable results on several diabetic retinopathy datasets, this study only used one family of teacher backbones, which were based on EfficientNet; this choice gave us a controlled setting for testing how much the ordinal-aware pruning and the EMD-based distillation contributed, while also keeping the differences from very different network architectures small. Since the design of the backbone can affect feature extraction, how the model learns, and how knowledge gets transferred, keeping the experiments within one architecture family made it easier to focus on checking the proposed compression strategy.

One thing this study did not do is test the framework on transformer-based or hybrid architectures, like Vision Transformer (ViT), Swin Transformer, ConvNeXt, and EfficientFormer. These models are quite different from regular convolutional networks in how they represent information, and they may react differently to pruning and distillation; so, it is still not clear how well ordinal-aware compression works across different backbone families.

But still, the main parts of OrdPrune-KD are mostly not tied to a specific architecture. The ordinal-aware pruning rule and the EMD-based distillation goal do not rely on operations that are only found in EfficientNet, so they could possibly be used on other convolutional or transformer-based models as well. In the future, we will look into how well this framework works on different backbone architectures, with a special focus on lightweight transformer models and on transferring ordinal knowledge across different architectures for medical image analysis.

### 5.4. Clinical Significance

Across all three datasets, distilled models generally maintain competitive high-risk sensitivity relative to the teacher model, which is critical for clinical screening applications. The IDRiD results are particularly notable: Distill Pruned 30% achieves a high-risk sensitivity of 0.938, meaning only 6.2% of severe proliferative cases would be missed.

## 6. Conclusions

In terms of performance, the proposed OrdPrune-KD method demonstrates stable and consistent effectiveness on multiple public benchmark datasets. On the APTOS 2019 dataset, the EfficientNet-B0-based student model achieves QWK = 0.9194 with only 4.01 M parameters (approximately 77% reduction), achieving comparable or slightly improved QWK scores under the current evaluation protocol relative to the EfficientNet-B4 teacher model (QWK = 0.9034), while using only 4.01 M parameters (approximately 77% parameter reduction). On the IDRiD dataset, even with only 319 training samples, the model combining distillation and 30% pruning achieves a QWK of 0.7585, demonstrating competitive performance relative to the teacher model (QWK = 0.7364) despite substantial model compression, and comprehensively outperforms the teacher model on AUC metrics for clinically critical grades G2, G3, and G4, demonstrating good small-sample generalization capability. In the Messidor-2 dataset, the B0 student model achieves a QWK only 2.4% lower than the teacher while reducing the parameters by 77%, and the high-risk sensitivity (0.939) remains close to the teacher model (0.970), indicating strong robustness in scenarios of cross-datasets.

In terms of efficiency, the MobileNetV3-based student model has an inference latency of only 3.6 ms on the GPU, achieving approximately 2.5× speedup compared to the teacher model while reducing FLOPs by 85.9%. This result demonstrates that the proposed method can maintain high diagnostic performance while significantly reducing computational overhead, with the capability to achieve real-time inference on mobile terminals and edge devices. This has important engineering application value for integrating intelligent diabetic retinopathy screening systems into mobile applications, mini-programs, and primary care devices.

In terms of clinical safety, the distilled lightweight models maintain high-risk sensitivity on all test datasets. On the IDRiD dataset, the model combining distillation and 30% pruning reaches a high-risk sensitivity of 0.938, with a miss rate for severe and proliferative diabetic retinopathy cases of approximately 6.2%. This result demonstrates that the proposed method can satisfy the basic requirements for high recall rates in clinical screening tasks while ensuring model lightweightness and efficiency, thereby having feasibility and safety for actual clinical deployment. Importantly, the proposed framework supports both architecture-preserving compression and ultra-lightweight heterogeneous deployment, enabling flexible adaptation to diverse clinical and edge-device deployment scenarios.

In summary, the OrdPrune-KD framework proposed in this paper demonstrates competitive performance across multiple datasets and achieves a good balance between model efficiency and clinically critical metrics. Future work will investigate the applicability of the proposed ordinal-aware compression framework to transformer-based and hybrid lightweight architectures for medical image analysis. Experimental results indicate that this method has potential application value for deployment in resource-constrained environments.

## Figures and Tables

**Table 1:** Dataset statistics and splits across the three benchmarks.

Dataset	Train	Validation	Test	Total
APTOS 2019	2930	366	366	3662
Messidor-2	976	244	524	1744
IDRiD	319	80	134	533

**Table 2:** Comprehensive evaluation on APTOS 2019. “Pruned 30%/50%/70%” denotes the target channel pruning ratios during structured pruning, while “Compress (%)” represents the actual parameter reduction relative to the original EfficientNet-B4 teacher model. All results use leak-free evaluation (thresholds optimized on the validation set).

Model	Params (M)	FLOPs (G)	Param Reduction (%)	QWK ↑	mAUC ↑	HR Sens. ↑	Latency (%) ↓
**Teacher Baseline**							
Teacher (EfficientNet-B4)	17.55	4.39	—	0.9034±0.0082	0.9050±0.0075	0.660±0.038	100
**Grade-Aware Pruning Only**							
Pruned 30%	9.37	2.35	46.6	0.8996±0.0091	0.9007±0.0083	0.640±0.041	95.6
Pruned 50%	6.16	1.56	64.9	0.8972±0.0095	0.8928±0.0092	0.620±0.043	93.4
Pruned 70%	3.95	1.01	77.5	0.8726±0.0118	0.8697±0.0109	0.600±0.045	95.6
**Pruning + EMD Ordinal Distillation (Ours)**							
Distill Pruned 30%	9.38	2.35	46.6	0.9154±0.0078	0.9006±0.0081	0.720±0.036	105.6
Distill Pruned 50%	6.16	1.56	64.9	0.9158±0.0079	0.8966±0.0087	0.640±0.040	69.7
Distill Pruned 70%	3.95	1.01	77.5	0.8992±0.0102	0.8849±0.0098	0.680±0.039	44.4
**Heterogeneous Distillation (Ours)**							
Distill→EfficientNet-B0	4.01	1.12	77.2	0.9194±0.0068	0.8842±0.0095	0.680±0.037	51.7
Distill→MobileNetV3	4.21	0.62	76	0.8871±0.0105	0.8827±0.0096	0.740±0.035	40.4

↑ indicates that a higher value represents better performance.

**Table 3:** Comprehensive evaluation on Messidor-2 (1744 real fundus images).

Model	Params (M)	FLOPs (G)	Param Reduction (%)	QWK ↑	mAUC ↑	HR Sens. ↑	Latency (%) ↓
**Teacher Baseline**							
Teacher (EfficientNet-B4)	17.55	4.39	—	0.8017±0.0095	0.8756±0.0078	0.969±0.0092	100
**Grade-Aware Pruning Only**							
Pruned 30%	8.71	2.21	50.4	0.6331±0.0142	0.8083±0.0115	0.909±0.0165	98.8
Pruned 50%	4.57	1.18	74	0.3564±0.0178	0.7210±0.0139	0.788±0.0210	91.1
Pruned 70%	1.71	0.46	90.3	0.1216±0.0135	0.6525±0.0148	0.030±0.0275	94.4
**Pruning + EMD Ordinal Distillation (Ours)**							
Distill Pruned 30%	8.72	2.21	50.3	0.7139±0.0128	0.8393±0.0102	0.849±0.0185	97.8
Distill Pruned 50%	4.57	1.18	74	0.4111±0.0165	0.7706±0.0128	0.606±0.0235	100
Distill Pruned 70%	1.72	0.46	90.2	0.2976±0.0189	0.7180±0.0137	0.606±0.0238	94.4
**Heterogeneous Distillation (Ours)**							
Distill→EfficientNet-B0	4.01	1.12	77.2	0.7778±0.0108	0.8494±0.0089	0.939±0.0125	50.5
Distill→MobileNetV3	4.21	0.62	76	0.7730±0.0112	0.8485±0.0091	0.909±0.0158	40.7

**Table 4:** Comprehensive evaluation on IDRiD (533 real fundus images—the most challenging low-data setting).

Model	Params (M)	FLOPs (G)	Param Reduction (%)	QWK ↑	mAUC ↑	HR Sens. ↑	Latency (%) ↓
**Teacher Baseline**							
Teacher (EfficientNet-B4)	17.55	4.39	—	0.7364±0.0175	0.8034±0.0148	0.729±0.0415	100
**Grade-Aware Pruning Only**							
Pruned 30%	8.71	2.21	50.4	0.6914±0.0198	0.7825±0.0162	0.938±0.0245	94.3
Pruned 50%	4.57	1.18	74	0.6652±0.0205	0.7454±0.0179	0.792±0.0372	91.1
Pruned 70%	1.71	0.46	90.3	0.3812±0.0236	0.6207±0.0204	0.583±0.0458	92.1
**Pruning + EMD Ordinal Distillation (Ours)**							
Distill Pruned 30%	8.72	2.21	50.3	0.7585±0.0168	0.8118±0.0142	0.938±0.0238	92.1
Distill Pruned 50%	4.57	1.18	74	0.6842±0.0192	0.7910±0.0159	0.854±0.0326	96.8
Distill Pruned 70%	1.72	0.46	90.2	0.4978±0.0224	0.6736±0.0191	0.542±0.0465	96.8
**Heterogeneous Distillation (Ours)**							
Distill→EfficientNet-B0	4.01	1.12	77.2	0.6805±0.0195	0.7364±0.0175	0.896±0.0284	47.9
Distill→MobileNetV3	4.21	0.62	76	0.6637±0.0208	0.7446±0.0178	0.729±0.0410	43.6

**Table 5:** Cross-dataset summary: Teacher vs. best distilled student on each benchmark. ΔQWK denotes the relative QWK difference between the best distilled student and the teacher.

Dataset	Model	Params (M)	FLOPs (G)	Param Reduction (%)	QWK	mAUC	HR Sens.	ΔQWK
APTOS (n=3362)	Teacher (B4)	17.55	4.39	—	0.9034	0.905	0.660	—
	Distill→B0	4.01	1.12	77.2	0.9194	0.884	0.680	+1.60%
Messidor-2 (n=17,742)	Teacher (B4)	17.55	4.39	—	0.8017	0.876	0.970	—
	Distill→B0	4.01	1.12	77.2	0.7778	0.849	0.939	−2.39%
IDRiD (n=533)	Teacher (B4)	17.55	4.39	—	0.7364	0.803	0.729	—
	Distill Pruned 30%	8.72	2.21	50.3	0.7585	0.812	0.938	+2.21%

**Table 6:** Fairness-controlled comparison on APTOS 2019 under identical output formulations.

Model	Training Strategy	QWK
EfficientNet-B0	Scratch	0.862±0.013
EfficientNet-B0	OrdPrune-KD	0.919±0.008
MobileNetV3	Scratch	0.834±0.016
MobileNetV3	OrdPrune-KD	0.887±0.010

**Table 7:** Ablation study results on APTOS 2019 (pruning ratio = 30%). Groups D/E use classification heads; Groups A–C use regression + threshold optimization.

Group	Method	Acc	QWK	mAUC	HR Sens.	Params
A	Teacher baseline	0.803	0.903	0.905	0.600	17.55 M
B	Uniform magnitude pruning	0.776	0.907	0.902	0.580	8.71 M
C	Grade-aware pruning (ours)	0.801	0.908	0.900	0.720	8.71 M
D	C + KL distillation	0.754	0.888	0.920	0.720	8.72 M
E	C + EMD distillation (ours)	0.757	0.889	0.904	0.760	8.72 M

**Table 8:** Comparison with published lightweight and DR grading methods on APTOS 2019.

Method	Year	QWK	ACC (%)	Params (M)	Notes
Published Methods (Five-class DR Grading)					
Kaggle Gold (EffNet-B5 Ensemble)	2019	0.936	–	–	Five-fold + TTA + ensemble
Dual-SwinOrd (Swin-T + PubMedCLIP)	2025	0.937	87.98	–	Vision-language dual-head
MSA-Net (Multi-Scale Attention)	2026	0.894	82.71	–	Five-fold CV
Ordinal Regression (ResNet-50)	2025	0.899	–	–	GC + CLAHE preprocessing
OrdPrune-KD (Ours)					
Teacher (EfficientNet-B4)	2026	0.903	80.3	17.55	Single-model baseline
OrdPrune-KD Pruned 30%	2026	0.915	77.9	9.38	46.6% compression
OrdPrune-KD→B0	2026	0.919	80.6	4.01	77% compression, 5.0 ms

**Table 9:** Computational efficiency comparison (APTOS 2019 results).

Model	Params	FLOPs	Param (M)	FLOPs	Latency	Speedup
Teacher (B4)	17.55 M	4.39 G	—	—	8.9 ms	1.0×
Distill Pruned 30%	9.38 M	2.35 G	46.6%	46.5%	9.6 ms	0.93×
Distill Pruned 50%	6.16 M	1.56 G	64.9%	64.5%	8.2 ms	1.09×
Distill Pruned 70%	3.95 M	1.01 G	77.5%	77.0%	6.4 ms	1.39×
Distill→B0	4.01 M	1.12 G	77.2%	74.5%	5.0 ms	1.78×
Distill→MNV3	4.21 M	0.62 G	76.0%	85.9%	3.6 ms	2.47×

## Data Availability

The original contributions presented in this study are included in the article. Further inquiries can be directed to the corresponding author.

## Abbreviations

The following abbreviations are used in this manuscript:

DR	Diabetic Retinopathy
QWK	Quadratic Weighted Kappa
EMD	Earth Mover’s Distance
KL	Kullback–Leibler Divergence
SWA	Stochastic Weight Averaging
FLOPs	Floating Point Operations
mAUC	Mean Area Under Curve
HR Sens	High-Risk Sensitivity
CDF	Cumulative Distribution Function
OrdCE	Ordinal Cross Entropy
